# Enhanced waste activated sludge digestion using a submerged anaerobic dynamic membrane bioreactor: performance, sludge characteristics and microbial community

**DOI:** 10.1038/srep20111

**Published:** 2016-02-01

**Authors:** Hongguang Yu, Zhiwei Wang, Zhichao Wu, Chaowei Zhu

**Affiliations:** 1 State Key Laboratory of Pollution Control and Resource Reuse, School of Environmental Science and Engineering, Tongji University, Shanghai 200092, P.R. China; 2 Chinese Research Academy of Environmental Sciences, Beijing 100012, P.R. China

## Abstract

Anaerobic digestion (AD) plays an important role in waste activated sludge (WAS) treatment; however, conventional AD (CAD) process needs substantial improvements, especially for the treatment of WAS with low solids content and poor anaerobic biodegradability. Herein, we propose a submerged anaerobic dynamic membrane bioreactor (AnDMBR) for simultaneous WAS thickening and digestion without any pretreatment. During the long-term operation, the AnDMBR exhibited an enhanced sludge reduction and improved methane production over CAD process. Moreover, the biogas generated in the AnDMBR contained higher methane content than CAD process. Stable carbon isotopic signatures elucidated the occurrence of combined methanogenic pathways in the AnDMBR process, in which hydrogenotrophic methanogenic pathway made a larger contribution to the total methane production. It was also found that organic matter degradation was enhanced in the AnDMBR, thus providing more favorable substrates for microorganisms. Pyrosequencing revealed that *Proteobacteria* and *Bacteroidetes* were abundant in bacterial communities and *Methanosarcina* and *Methanosaeta* in archaeal communities, which played an important role in the AnDMBR system. This study shed light on the enhanced digestion of WAS using AnDMBR technology.

Waste activated sludge (WAS) is generated during wastewater biological treatment process, and potentially a secondary pollutant if not properly coped with. WAS treatment and disposal accounts for up to 50% of the operating costs in wastewater treatment plants (WWTPs), challenging the municipal wastewater management worldwide^1^,^2^. For WAS treatment, anaerobic digestion (AD) is attractive because of its advantages such as sludge amount reduction, biogas production and pathogen destruction^2^. However, some drawbacks exist in conventional AD (CAD) processes hindering their wide-spread applications. For example, sludge thickening is needed prior to AD process in order to reduce WAS volume. Besides, hydraulic retention time (HRT) is identical to solid retention time (SRT), leading to a larger volume of digester and the non-flexible operation of CAD processes. On the other hand, WAS, particularly in biological treatment systems with long SRTs, presents relatively poor anaerobic biodegradability compared to primary sludge due to the accumulation of cellular residues and suspended inert materials^3^,^4^, which also negatively affects AD performance.

In order to improve AD performance, some high-rate AD processes, such as expanded granule sludge blanket (EGSB)^5^ and anaerobic membrane bioreactor (AnMBR)^1^,^6^, have been proposed. For EGSB technology, sludge granulation is complex and demanding, and WAS, unlike wastewater, may influence the performance of anaerobic granules^5^. AnMBR process prevails over CAD process in terms of footprint reduction, concurrent thickening and digestion, and decoupling HRT from SRT^1^. The efficient solid/liquid separation of membranes well retains microorganisms and thus enhances pollutant degradation^7^. Recently, AnMBR systems with microfiltration/ultrafiltration (MF/UF) membranes have been applied to WAS digestion. Dagnew *et al.*^8^ employed external tubular membranes to treat polymer-dosed thickened WAS (total solids 17.0 g/L) in a pilot-scale anaerobic digester, and observed about 48% of volatile solids reduction rate under HRT 15 d and SRT 30 d. Similar volatile solids destruction rate (45–51%) was reported by Xu *et al.*^9^ using an external AnMBR system for the digestion of thickened WAS at membrane flux 1.3–3.5 L/(m^2^ h). However, the major drawbacks in the AnMBR processes are low membrane flux of MF/UF membranes and high membrane fouling rate^6^. Moreover, the external membrane configuration results in large energy consumption since fouling is controlled via high circulation velocities, which may also inhibit methanogenic activity due to the intense pump shear^6^,^10^.

In AnMBR processes, cake formation is a major contributor to membrane fouling and detrimental to the filtration performance^11^. However, cake formation on membrane surfaces can be beneficial to the filtration operation on the other side, which is termed dynamic membrane (DM) filtration^12^,^13^,^14^. The *in-situ* deposited cake layer, namely a DM layer, accomplishes solid-liquid separation rather than the support material. Therefore, supporting materials can be made of meshes, fabrics and other cheap materials instead of expensive MF/UF membranes^13^. Moreover, physical cleaning is adequate to restore DM permeability, which spares chemical cleaning reagent during long-term operation^15^. With the combination of DM technology, anaerobic dynamic membrane bioreactor (AnDMBR) process seems to address the shortages of AnMBR systems.

AnDMBRs have been successfully applied to municipal wastewater^16^, high-strength synthetic wastewater^17^ and landfill leachate^18^ treatment. However, studies on AnDMBR technology for WAS treatment are very limited. In our previous publication, an AnDMBR system for WAS digestion was successfully started-up^19^. Compared to wastewater treatment processes, WAS digestion systems are operated under much higher solid concentrations, which may challenge the performance of DM filtration. To date, there is an obvious lack of a systematic study on the performance of an AnDMBR for WAS digestion.

In the present work, therefore, we aimed to investigate the long-term performance of an AnDMBR system for WAS treatment. The objectives of this study were: (1) to compare the digestion performance between AnDMBR and CAD; (2) to characterize the properties of digested sludge; and (3) to elucidate the mechanisms through biochemical and microbial analyses.

## Results and Discussion

### Digestion performance

Anaerobic biodegradability of the feed sludge is a crucial factor affecting the AD performance. In this study, biochemical methane potential (BMP) tests were conducted for identifying WAS anaerobic biodegradability^4^,^20^. The maximum methane production of WAS in our study was 199.5 ± 6.4 mL/gVSS_added_ (See Supplementary Fig. S1). Compared to other BMPs of WAS (206–427 mL/gVSS_added_) in available literature^4^, the BMP value in the present work was at low level, indicating a relatively poor anaerobic biodegradability of the feed WAS.

**Figure 1:**
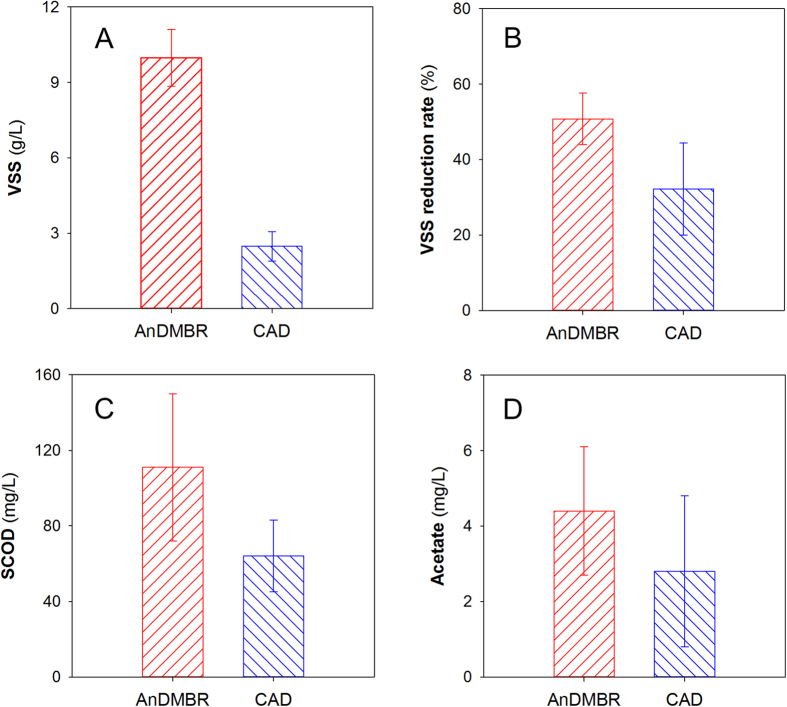
Performance of the AnDMBR and CAD processes. (**A**) VSS concentrations in reactors, (**B**) VSS reduction rate, (**C**) SCOD concentrations in reactors and (**D**) acetate concentrations in reactors. Error bars represent standard deviations (*n* = 30 for VSS, VSS reduction rate and SCOD, and *n* = 19 for acetate).

**Figure 2:**
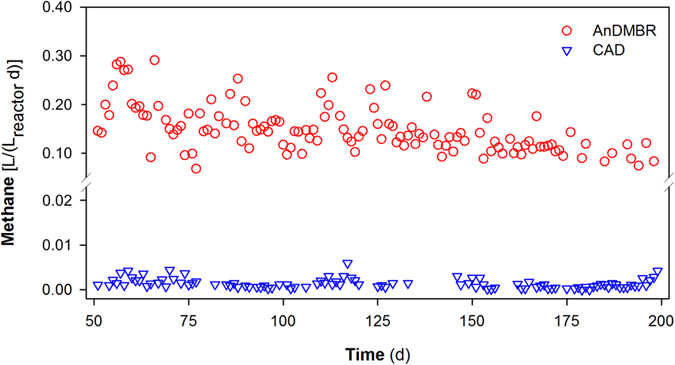
Methane production in the AnDMBR and CAD processes.

The performance of AnDMBR and CAD was monitored for 200 d (Figs 1 and 2). As shown in Fig. 1A, VSS concentration in the AnDMBR was 4.0 times that in the CAD process, implying that the AnDMBR had the function of sludge thickening. Meanwhile, a 50.8 ± 6.8% of volatile suspended solids (VSS) reduction rate was achieved in the AnDMBR, higher than that in the CAD (Fig. 1B), indicating the improved VSS destruction in the system. The results demonstrated that the AnDMBR process could achieve concurrent WAS thickening and digestion^8^. The soluble chemical oxygen demand (SCOD) concentration in the AnDMBR was 1.7 times that of CAD (Fig. 1C), showing that the AnDMBR might enhance sludge hydrolysis^21^. Volatile fatty acid (VFA) analysis demonstrated that acetate was the most predominant component, accounting for more than 90% of total VFAs. However, acetate concentrations in both systems were low (Fig. 1D), suggesting that the produced VFAs were rapidly utilized for methane production. After sludge digestion, large amounts of ammonia were produced (See Supplementary Fig. S2). The ammonium concentration in the AnDMBR was 172.7 mg/L on average, slightly higher than that in the CAD. However, it is still lower than the threshold value of 200 mg/L that could inhibit AD process as reported elsewhere^22^.

As shown in Fig. 2, methane production of the AnDMBR was 0.15 ± 0.05 L/(L_reactor_ d), much higher than that in the CAD. The specific methane production based on removed VSS for the AnDMBR was 0.27 ± 0.07 L/gVSS_removed_, which is also much higher than that in the CAD (0.02 ± 0.02 L/gVSS_removed_). In order to explain the reasons for the enhanced methane production in the AnDMBR, specific methanogenic activity (SMA) tests for sludges in the two systems were carried out. Acetate and H_2_/CO_2_ were chosen as the substrates in SMA tests to evaluate the activities of acetoclastic and hydrogenotrophic methanogens, respectively. As shown in Supplementary Table S2, both of the SMA values based on acetate and H_2_/CO_2_ for the AnDMBR were higher than those for the CAD. SMA values might be related to relative abundances of methanogens, which will be discussed in the Microbial analyses section. The higher methanogenic activity of biomass in the AnDMBR process validated the enhanced methane production of the AnDMBR. In AnDMBRs, a high volumetric solid load can be achieved due to the decoupling of HRT from SRT. At the same SRT operation, the solid load of the AnDMBR system under a shortened HRT was five times that of the CAD process (0.17 kgVSS/m^3^ d). In this way, sufficient substrates were provided for digestion in the system, contributing to the improvement of methane production. In addition, biogas recirculation in the AnDMBR not only controlled membrane fouling (as discussed in the following section), but also provided additional mixing effect^23^, which facilitated the interactions between feed sludge and active biomass and intensified mass transfer to further improve WAS digestion performance.

Besides the enhanced total methane production, high methane (CH_4_) content in the biogas was also observed in the AnDMBR. In the system, the biogas contained 72.0 ± 8.2% of CH_4_, higher than AD processes as reported in literature^2^. Therefore, a larger proportion of CH_4_ in the biogas from the AnDMBR system indicated higher energy recovery potential. The high methane content in biogas of AnDMBR may be closely related to methanogenic pathways. In order to identify them, stable carbon isotopic signatures were analyzed in our study (Table 1). Methanogenic pathways can be estimated by the apparent fractionation factor α_c_, and a higher α_c_ value indicates a larger contribution of the hydrogenotrophic methanogenic pathway to total methane production. Usually α_c_ > 1.065, α_c_ < 1.025 and α_c_ around 1.045 represent for hydrogenotrophic methanogenesis, acetoclastic methanogenesis, and the combination of the two pathways, respectively^24^,^25^. It can be inferred from Table 1 that both two AD processes contained the combined methanogenesis, but hydrogenotrophic pathway played a more important role in the AnDMBR, resulting in the higher CH_4_ and lower CO_2_ content in the system.

### DM filtration performance

Dynamic layer formation holds the key to the filtration performance in AnDMBRs^11^,^13^; however, an over-growth of DM layer leads to a rapid increase in trans-membrane pressure (TMP). In order to control the rapid growth of DM, biogas sparging with a sparging intensity of 37.5 m^3^/(m^2^ h), which falls within a typical range of 17.6–65 m^3^/m^2^ h in AnMBRs^26^, was adopted in the present work. Our preliminary studies showed that continuous biogas sparging significantly affected DM formation, resulting in poor effluent quality (effluent turbidity >1000 NTU). Therefore, intermittent biogas sparging mode was chosen to facilitate the formation and control of DM layer in the long-term operation. Moreover, intermittent biogas recirculation mode (120-min off and 20-min on) spared the biogas recirculation energy consumption by 85.7% in comparison with continuous sparging at the same biogas sparging rate.

**Figure 3:**
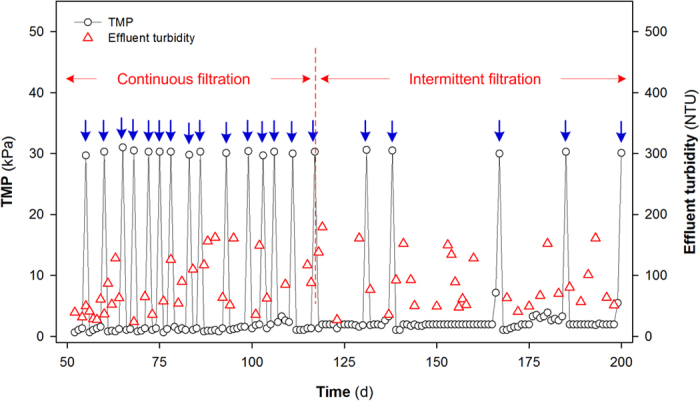
TMP and effluent turbidity variations of the AnDMBR. The blue downward arrow indicates where physical cleaning was carried out.

The changes of trans-membrane pressure (TMP) as a function of operation time are shown in Fig. 3. During the long-term operation, two permeation modes were adopted in the AnDMBR, i.e., continuous filtration and intermittent filtration (10-min suction and 2-min pause). In both filtration modes, TMP profile exhibited an obvious two-stage phenomenon, including an initial slow TMP increase and a subsequent short-period rapid TMP rise^18^. The sudden increases of TMP values might be due to the over-growth and fast compaction of DM layer^16^, especially with much higher solid concentrations in the sludge digestion system than wastewater treatment processes. In the AnDMBR, larger particles in the mixed liquor were effectively rejected by the DM layer in both filtration modes (Supplementary Fig. S3). The effluent turbidity for the two filtration modes were 84.4 ± 60.8 NTU and 98.0 ± 66.6 NTU, respectively, which showed no significant difference in effluent turbidity (*p* = 0.40 in *t*-test). However, intermittent filtration exhibited a longer operating cycle (16.6 ± 8.0 d) compared to continuous filtration (4.3 ± 1.3 d) (Fig. 3), demonstrating its advantage in controlling the rapid growth of DM. This might be attributed to the fact that under the intermittent filtration mode, part of membrane foulants could diffuse away from the membrane surface due to concentration gradient and surface shear forces when pump suction was paused^27^.

### Sludge characteristics

**Figure 4:**
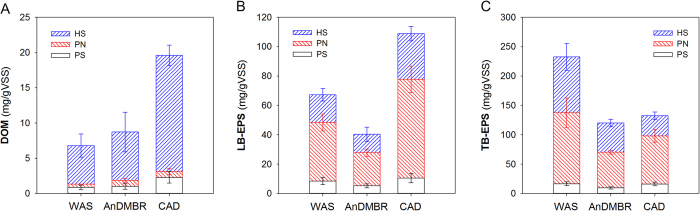
Extracellular organic matter distribution of sludge samples. (**A**) DOM; (**B**) LB-EPS; (**C**) TB-EPS. PS, PN and HS denote polysaccharides, proteins and humic substances, respectively.

In AD process, sludge hydrolysis leads to the rupture of cell walls and the release of extracellular polymeric substances (EPS), which provides soluble organic substrates, such as dissolved organic matter (DOM), for acidogenic microorganisms^2^. Therefore, DOM and bound EPS contents in sludge flocs are significant indicators to characterize AD process. Distribution of three fractions, i.e., DOM, loosely bounded EPS (LB-EPS) and tightly bounded EPS (TB-EPS), is depicted in Fig. 4. Both LB-EPS and TB-EPS contents for various sludges followed the order of AnDMBR sludge < CAD sludge < WAS. The EPS content difference between AnDMBR sludge and WAS was larger than that between CAD sludge and WAS, showing that the AnDMBR achieved an enhanced EPS destruction. Meanwhile, DOM contents in the AnDMBR sludge were also lower than those in the CAD sludge, suggesting that the produced DOM originated from EPS and intracellular polymeric substances, being electron-donors^28^, was efficiently utilized *in situ* to generate biogas. The improved degradation of extracellular organic matter might be explained by the higher abundance of functional bacteria in the AnDMBR process, which will be discussed in the Microbial analysis section.

The fluorescence properties of DOM samples were also explored using excitation–emission matrix (EEM) with fluorescence regional integration (FRI) analysis (Supplementary Fig. S4). Substrates from Region II and IV appear high biodegradability, while those from Region III and V exhibit low biodegradability^29^. Higher percentages of Region II and IV, along with lower percentages of Region III and V, were observed in the DOM fraction of the AnDMBR compared to the CAD, indicating that the AnDMBR system provided more favorable substrates for subsequent metabolism of anaerobic microbes. This also partially explains why the AnDMBR had an enhanced methane production.

In WAS treatment, the following step after AD is normally dewatering. In the present work, sludge dewatering properties were compared based on normalized capillary suction time (CST_n_)^30^. As shown in Supplementary Fig. S5, CST_n_ values of sludge samples in the AnDMBR and the CAD showed no statistical difference (*p* = 0.65 in *t*-test), implying that the AnDMBR sludge exhibited similar dewatering properties to the CAD sludge. Furthermore, we analyzed the relations between DOM compositions and CSTn values, and found that protein contents in DOM were significantly related to CST_n_ values (Supplementary Fig. S6), which indicated the notable influence of DOM protein contents on sludge dewaterability^31^. Similar protein amounts in the DOM fractions of the two AD systems (*p* = 0.95 in *t*-test, Fig. 4A) might explain the CST_n_ results of the digested sludge.

### Microbial analyses

In order to elucidate the microbial communities for WAS digestion, 6 libraries in total were constructed for the bacteria and archaea domains for the three sludge samples. As listed in Supplementary Table S1, the coverage values of sludge samples were larger than 0.98 in both bacterial and archaeal community, implying that the most common phylogenetic groups were detected in our libraries^32^. Chao and Shannon indices exhibited decreasing bacterial diversities and increasing archaeal diversities during WAS digestion.

**Figure 5:**
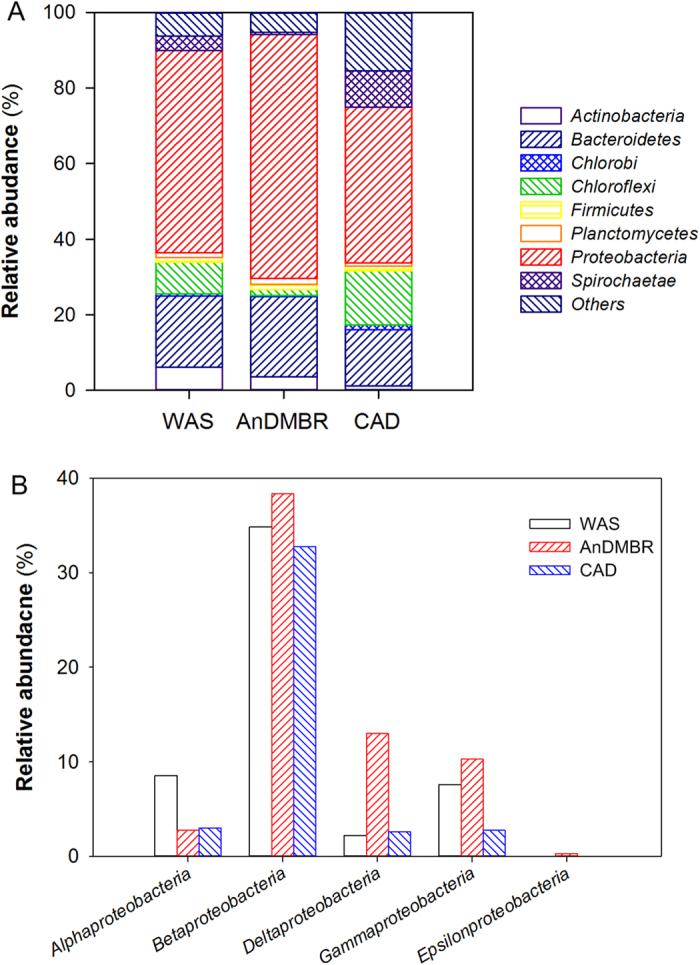
Bacterial communities. (**A**) Phylum level; (**B**) subdivisions of *Proteobacteria* at class level. Relative abundance is defined as the number of sequences affiliated with that taxon divided by the total number of sequences per sample (%). Phyla accounting for less than 1% of relative abundance are regarded as others.

In bacterial communities, *Proteobacteria* and *Bacteroidetes* were the two most predominant phyla in the digested sludge samples (Fig. 5A), which are also reported in other AD systems^33^,^34^. *Proteobacteria* are able to degrade a wide range of macromolecules^33^; *Bacteroidetes*, known to be proteolytic bacteria, are involved in protein degradation and able to ferment amino acids to acetate^32^. *Proteobacteria* and *Bacteroidetes* accounted for a larger proportion in the AnDMBR process, which might explain its improved organic matter degradation (Fig. 4). Among these phyla, *Proteobacteria* were the highest-ranked, and distributions of the five subdivisions (i.e., *alpha-*, *beta-*, *gamma-*, *delta-*, and *epsilon-*) are shown in Fig. 5B. *Betaproteobacteria* were the most predominant class in the digested sludge samples, which are reported to compose the core group in organic matter degradation^32^. Higher relative abundance of *Betaproteobacteria* in the AnDMBR might validate its enhanced degradation performance. Moreover, *Betaproteobacteria* are also reported to be predominant in propionate-, butyrate-, and acetate-utilizing microbial communities^35^, which might be related to the low VFA concentrations in both AD systems (Fig. 1D). On the other hand, bio-hydrogen producing bacteria, such as genera of *Rhodobacter* belonging to *Alphaproteobacteria*^36^, were observed in the present study, suggesting that versatile methanogenic pathways, e.g., hydrogenotrophic methanogenesis, might occur in the AD processes.

**Figure 6:**
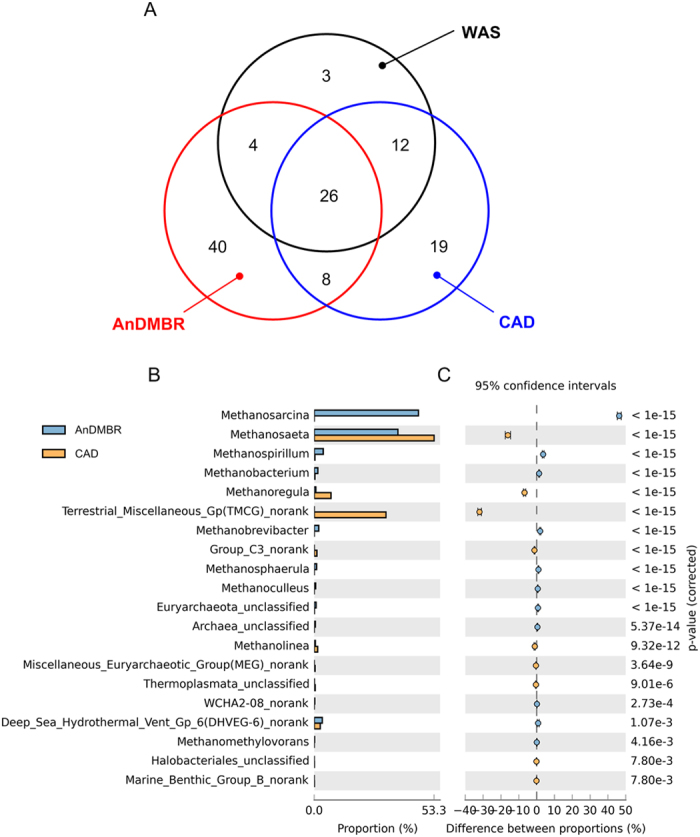
Archaeal communities. (**A**) Venn diagram based on OTUs (3% distance); (**B**) relative abundances of the phylogenetic genera; (**C**) statistical analysis of the differences between relative abundances. Relative abundance is defined as the number of sequences affiliated with that taxon divided by the total number of sequences per sample (%).

In order to illustrate the similarities of various archaeal communities, Venn analyses were conducted on the three sludge samples based on OTUs at a dissimilarity level of 0.03 (Fig. 6A). The total number of observed OTUs was 112, with 26 OTUs (accounting for 23.2%) commonly shared by the three sludge samples. Compared to the CAD sludge, the AnDMBR sludge showed a larger number of unique OTUs, and shared a smaller number of OTUs with WAS. It seemed that archaeal communities altered more significantly in the AnDMBR system. In addition, a pairwise statistical comparison between the two AD processes was carried out at the genus level (Fig. 6B,C). Two major methanogenic genera in the AnDMBR were *Methanosarcina* and *Methanosaeta*. *Methanosarcina* accounted for 46.4% of the total reads on the genus level in the AnDMBR. *Methanosaeta* were the second most abundant genus in the AnDMBR, while they were the most abundant genus in the CAD. In comparison, the AnDMBR contained more abundant *Methanosarcina* and less abundant *Methanosaeta* than the CAD in a statistically notable way. *Methanosarcina* are reported as robust methanogens which can tolerate stressors such as high levels of ammonium and salt, pH shock and organic overloading^37^. Therefore, *Methanosarcina* might overwhelm other vulnerable genera during the long-term operation of the AnDMBR system. Besides, total relative abundances of *Methanosarcina* and *Methanosaeta* in the AnDMBR were higher than those in the CAD, which is consistent with the SMA results (Supplementary Table S2).

The genera of *Methanosarcina* and *Methanosaeta* consume different kinds of substrates for methanogenesis^38^. *Methanosarcina* are able to utilize a wide variety of organic substrates such as acetate, H_2_, CO_2_, methanol and formate^37^, which supported the occurrence of combined methanogenic pathway (acetoclastic and hydrogentrophoc) in the AnDMBR. On the other hand, *Methanosaeta* are acetoclastic methanogens, of which the higher relative abundance might lead to the dominance of acetoclastic methanogenesis in the CAD. Archaeal communities of the two AD systems (Fig. 6B,C) correspond with the methanogenic pathway identification as shown in Table 1.

### Applicability of AnDMBR system

In the present work, the AnDMBR process exhibited enhanced WAS digestion performance over the CAD process. Energy balance analyses of the two AD systems (Supplementary Information Section 2 and Fig. S7) showed that compared to the CAD process, about 37.3% of net energy demand was reduced, indicating the improved energy-efficiency of the AnDMBR system. For example, in a full-scale wastewater treatment plant with daily excess sludge production of 2000 kg (dry sludge), use of the AnDMBR technology instead of the CAD process could achieve the substantial improvement of sludge digestion and spare ~6.6 × 10^5^ kWh of annual net energy consumption (Supplementary Information Section 2). However, further improvements are required prior to practical applications of the AnDMBR technology in the following aspects. The largest challenge is the negative net energy production (Supplementary Fig. S7). Heating accounts for the largest proportion of total energy consumption due to the temperature difference between reactor and feed sludge. In order to address the challenge, 65% of the recovered energy via methane combustion that is given off as heat^39^ can be utilized to compensate the heating energy consumption. Attempts can be also made to the optimization of AnDMBR operation under ambient temperature conditions to reduce the heating energy demand. Apart from the energy considerations, AD pretreatment methods such as ultrasound can be adopted to improve the anaerobic degradability of feed sludge prior to the AnDMBR process^40^.

In summary, we investigated the long-term performance of a submerged AnDMBR system to treat WAS with poor anaerobic biodegradability. VSS reduction rate of 50.8% and specific methane production of 0.27 L/gVSS_removed_ were achieved. High-quality biogas with 72.0% CH_4_ content was produced from the system, attributed to a larger contribution of the hydrogenotrophic methanogenic pathway as revealed by stable isotopic signature analysis. The AnDMBR system exhibited effective filtration performance by using intermittent biogas sparging and intermittent filtration modes. Moreover, the AnDMBR promoted extracellular organic matter degradation and provided more favorable substrates than the CAD. The digested sludge in the AnDMBR exhibited similar dewaterability to that in the CAD. Pyrosequencing revealed that higher relative abundances of *Proteobacteria* and *Bacteroidetes* in bacterial communities were observed in the AnDMBR process, which might be related to its enhanced organic matter degradation. In archaeal communities, *Methanosarcina* and *Methanosaeta* were the major genera responsible for methane production in the AnDMBR, in accordance with the methanogenic pathway identification. The enhanced WAS digestion performance in AnDMBRs might be due to decoupling HRT from SRT, biogas recirculation, high organic solids load and the induced unique microbial community.

## Methods

### Experimental setup and operation

**Figure 7:**
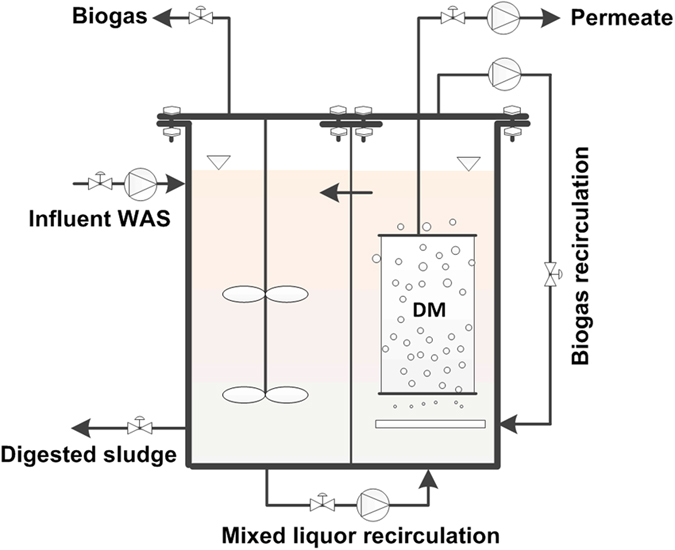
Schematic of the AnDMBR process.

The AnDMBR system for direct treatment of WAS is shown in Fig. 7. Excess sludge from the Quyang WWTP (Shanghai, China, 31.3 °N 121.5 °E) was used as the influent after passing through a mesh (pore size = 0.9 mm). The characteristics of the influent WAS are as follows: VSS 3.47 ± 0.82 g/L, SCOD 30 ± 17 mg/L, acetate 3.5 ± 2.4 mg/L, ammonium 4.9 ± 5.2 mg/L, and CST_n_ 3.3 ± 0.5 s L/gTSS. The liquor level in the system was controlled using an elevated influent tank. The AnDMBR system consisted of a completely mixed anaerobic digester (effective volume of 67 L) coupled with a submerged anaerobic dynamic membrane reactor (effective volume of 2 L). The configuration facilitated convenient membrane cleaning and replacement in the membrane zone while maintaining the main digester strictly anaerobic at all times. HRT and SRT of the system were 5 d and 20 d, respectively. A flat-sheet dynamic membrane module was mounted in the membrane zone, which was made of Dacron mesh (pore size = 39 μm). A peristaltic pump was installed to recycle sludge from the anaerobic digester to the dynamic membrane zone at a recirculation ratio of 300%, and another peristaltic pump was used to withdraw permeate from the dynamic membrane module. The effluent flow rate was controlled by a flowmeter. Trans-membrane pressure (TMP) was monitored using a pressure gauge on a daily basis, and an average value was reported. Biogas production was measured according to the volume of biogas collected in the wetted gas collector (LMF-1, Duoyuan Instrument Technology Co., Ltd., China), in which the gas pressure was maintained at a pressure of 1 atm. Electric heaters controlled by temperature sensors were used to maintain the temperature of the system at 35 ± 2 °C. Biogas was recycled using a diaphragm gas pump (KNF, Germany) under an intermittent working mode (120-min off and 20-min on) to scour membrane surfaces for fouling control, and the biogas sparging rate per unit projected area of the riser zone was controlled at 37.5 m^3^/(m^2^ h). The dynamic membrane module was operated at an instant flux of ~15 L/(m^2^ h). In our study, two operation modes for effluent suction pump were applied. From 51 d to 117 d, continuous filtration was applied with the membrane area of 0.038 m^2^. From 118 d to 200 d, intermittent filtration (10-min suction and 2-min pause) was adopted. In order to maintain the same HRT, the membrane area was increased to 0.046 m^2^. Physical cleaning was conducted for the dynamic membrane when TMP increased to 30 kPa.

Meanwhile, a lab-scale conventional anaerobic digestion (CAD) reactor with effective volume of 5 L was operated as control test. The stirring speed and temperature were set at 50 rpm and 35 ± 2 °C, respectively, to keep the same condition as the AnDMBR. The above-mentioned WAS was also used as the feed sludge of the CAD. In CAD processes, HRT and SRT are identical^1^,^2^. Therefore, 5 d and 20 d of SRT (HRT) were both needed to set for comparison of AnDMBR. However, it has been reported that retention times shorter than 5 d are insufficient for a stable digestion in CAD, and digestion performance increased with an increase of SRT when SRT is shorter than 20 d^2^. Thus, we chose 20 d as the SRT (HRT) of the CAD for achieving better digestion performance. The two reactors were subject to acclimation for 50 d prior to the experiments of this work.

### Analyses and calculations

#### Biochemical methane potential (BMP) and specific methanogenic activity (SMA) tests

In order to characterize the anaerobic biodegradability of WAS, BMP tests were conducted according to the protocol reported by Angelidaki *et al.*^20^. The influent WAS and AnDMBR sludge samples were chosen as substrate and inoculum, respectively, and the inoculum to substrate VSS ratio was 1^41^. BMP tests were carried out in triplicate at 35 ± 2 °C. Meanwhile, SMA was measured to evaluate the methanogenic ability of biomass in the AnDMBR and CAD. Acetate and H_2_/CO_2_ were used as the substrates, respectively. SMA tests were performed in triplicate according to our previous study^19^.

#### Extraction and determination of extracellular organic matter

Extracellular organic matter was divided into dissolved organic matter (DOM) and bound extracellular polymeric substances (EPS) fractions. DOM was extracted based on our previous study^42^, while bound EPS, including loosely bound EPS (LB-EPS) and tightly bound EPS (TB-EPS), were extracted according to Han *et al.*^43^. Three main components of DOM and EPS, i.e., polysaccharides, proteins, and humic substances^44^, were determined and normalized to the solids content of sludge samples. Polysaccharides were determined by the anthrone method with glucose as the standard reference^45^, while proteins and humic substances were measured using the modified Lowry methods using bovine serum albumin and humic acid as standard references, respectively^44^.

In addition, three-dimensional excitation emission matrix (EEM) fluorescence spectra were obtained using a luminescence spectrometry (F-4500 FL spectrophotometer, HITACHI, Japan). After partially removing Rayleigh and Raman scatters, fluorescence regional integration (FRI) method was applied to calculate the percentages of five excitation–emission regions^46^,^47^.

### Microbiology analyses

In this study, 454 high-throughput pyrosequencing was employed to reveal the microbial community structures of different systems. Sludge samples of influent, AnDMBR and CAD were collected on day 180 when the reactors were deemed to have achieved their steady-state operation after running for more than 3 times SRT. Microbial analyses were carried out according to our previous study^19^. The pyrosequencing procedures were documented in Supplementary Information. A pairwise statistical comparison of the taxonomy between the two samples was carried out using STAMP (two-sided Welch’s *t*-test on the alpha level of 0.05)^48^.

#### Stable carbon isotopic fractionation

In anaerobic digestion, methanogenic pathways can be quantified by stable carbon isotopic fractionation^24^. After steady-state operation of the reactors was reached, gas samples of AnDMBR and CAD were collected by gas sampling bags to measure the stable isotope signatures of CH_4_ (δCH_4_) and CO_2_ (δCO_2_). The isotopic analyses were carried out using an isotope ratio mass spectrometer (Isoprime, GV, U.K.) linked to a gas chromatograph (6890N, Agilent Technologies, U.S.A.) with a CP-poraplot Q column (25 m × 0.32 mm × 20 μm) according to the protocol reported elsewhere^49^.

The apparent carbon fractionation factor (α_c_) was calculated using equation (1) 24:


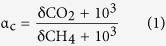


where δCH_4_ and δCO_2_ are the ^13^C isotope signatures of total CH_4_ and CO_2_.

#### Other analytical and calculation methods

Analytical parameters of sludge samples, such as soluble chemical oxygen demand (SCOD), ammonium nitrogen (NH_4_^+^-N), total suspended solids (TSS) and volatile suspended solids (VSS), were determined according to the *Standard Methods*^50^. Effluent turbidity was tested by a portable turbidity meter (2100Q, Hach Company, USA). Gas composition (CH_4_ and CO_2_) was measured using a gas chromatography (6890N, Agilent, U.S.) equipped with a thermal conductivity detector (TCD). Volatile fatty acid (VFA) compositions (mainly acetate in our study) were analyzed via a gas chromatography (6890N, Agilent, U.S.) equipped with a flame ionization detector (FID). Capillary suction time (CST) was tested by a capillary suction timer (Model 304M CST, Triton Electronics Ltd., England). Since CST values are related to biomass concentrations, in order for fair comparison, CST values were divided by the TSS concentration and expressed as normalized CST (CST_n_) with a unit of s L/gTSS^31^. An unpaired two-tailed *t*-test was applied to compare differences between two data groups (except for microbial data) on the alpha level of 0.05 using SigmaPlot (Version 11.0, Systat Software, Inc., U.S.).

VSS reduction rates of the reactors were calculated according to equation (2):


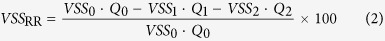


where *VSS*_RR_ is the VSS reduction rate (%), and *VSS*_0_, *VSS*_1_ and *VSS*_2_ are the VSS concentrations of the feed WAS, digested sludge and membrane permeate, respectively (g/L), and *Q*_0_, *Q*_1_ and *Q*_2_ are the flow rates of the feed WAS, digested sludge and membrane permeate, respectively (L/d). For the CAD reactor, *VSS*_2_ and *Q*_2_ values are equal to zero.

## Additional Information

**How to cite this article**: Yu, H. *et al.* Enhanced waste activated sludge digestion using a submerged anaerobic dynamic membrane bioreactor: performance, sludge characteristics and microbial community. *Sci. Rep.* **6**, 20111; doi: 10.1038/srep20111 (2016).

## Supplementary Material

Supplementary Information

## Figures and Tables

**Table 1:** Stable isotopic indicators of the two AD systems^a^.

Parameters (unit)	AnDMBR	CAD
δCH_4_ (%)	−51.50 ± 0.14	−44.80 ± 0.28
δCO_2_ (%)	1.20 ± 0.14	−4.25 ± 0.07
α_c_	1.056 ± 0.001	1.042 ± 0.001

^a^Data are given as average value ± standard deviation (n = 4).
