# Supplementary material for: Enhanced waste activated sludge digestion using a submerged anaerobic dynamic membrane bioreactor: performance, sludge characteristics and microbial community
Source: Sci Rep. 2016 Feb 1;6:20111. doi: 10.1038/srep20111 (PMC4735592; doi:10.1038/srep20111)
Supplement: Supplementary Information [file srep20111-s1.doc]

Supporting information

**Enhanced waste activated sludge digestion using a submerged anaerobic dynamic membrane bioreactor: performance, sludge characteristics and microbial community**

Hongguang Yu^1^, Zhiwei Wang^1^,*, Zhichao Wu^1^, Chaowei Zhu^2^

^1^State Key Laboratory of Pollution Control and Resource Reuse, School of Environmental Science and Engineering, Tongji University, Shanghai 200092, PR China

^2^Chinese Research Academy of Environmental Sciences, Beijing 100012, P.R. China

*Corresponding author. Tel/Fax: +86(21)65980400; E-mail address: zwwang@tongji.edu.cn (Z.W. Wang)

**Section 1 Microbiology analyses**

**Section 1.1 DNA extraction and PCR amplification**

Sludge samples of influent, AnDMBR and CAD were collected on day 180, and stored at -20 °C before further analysis.

Microbial DNA was extracted from sludge samples using an E.Z.N.A.® Soil DNA Kit (Omega Bio-tek, Norcross, GA, U.S.) according to manufacturer’s protocols. The V1-V3 and V3-V5 regions of the bacteria and archaea 16S ribosomal RNA gene were amplified by polymerase chain reaction (95 °C for 2 min, followed by 25 cycles at 95 °C for 30 s, 55 °C for 30 s, and 72 °C for 30 s and a final extension at 72 °C for 5 min) using primers 27F (5’-AGAGTTTGATCCTGGCTCAG-3’) / 533R (5’-TTACCGCGGCTGCTGGCAC-3’) and Arch344F (5’-ACGGGGYGCAGCAGGCGCGA-3’) / Arch915R (5’-GTGCTCCCCCGCCAATTCCT-3’), respectively. PCR reactions were performed in a 20 μL mixture containing 4 μL of 5 × FastPfu Buffer, 2 μL of 2.5 mM dNTPs, 0.8 μL of each primer (5 μM), 0.4 μL of FastPfu Polymerase, and 10 ng of template DNA.

**Section 1.2 454 pyrosequencing**

After purification using the AxyPrep DNA Gel Extraction Kit (Axygen Biosciences, Union City, CA, U.S.) and quantification using QuantiFluor™ -ST (Promega, U.S.), a mixture of amplicons was used for pyrosequencing on a Roche 454 GS FLX+ Titanium platform (Roche 454 Life Sciences, Branford, CT, U.S.) according to standard protocols [^1^](#_ENREF_1). The raw reads were deposited into the NCBI Sequence Read Archive (SRA) database (Accession Number: SRA303322).

**Section 1.3 Processing of pyrosequencing data**

Raw data processing and denoising were performed with the QIIME pipeline (version 1.17, <http://qiime.org/>) [^2^](#_ENREF_2). In total, 21887 and 30683 high-quality sequences were produced with an average length of 422 and 471 bp per sequence for bacteria and archaea, respectively (Table S1). In order for fair comparison at the same sequencing depth, samples were rarefied to 4046 and 8609 reads per sample for bacteria and archaea, respectively. Reads sharing 97% nucleotide sequence identity were grouped into operational taxonomic units (OTUs) using UPARSE pipeline (version 7.1, <http://drive5.com/uparse/>), and uchime (version 4.2.40) was used to filter out chimeras [^3^](#_ENREF_3). For each cluster file, α-diversity (Chao and Shannon indices) was calculated in MOTHUR (version v.1.30.1, [http://www.mothur.org](http://www.mothur.org/)). Taxonomy of the representative sequences from each OTU was conducted using the RDP Classifier (version 2.2, <http://sourceforge.net/projects/rdp-classifier/>) and Silva database (Release 115, [http://www.arb-silva.de](http://www.arb-silva.de/) ) with a set confidence threshold of 70% [^4^](#_ENREF_4).

**Section 2 Energy balance analysis**

The overall energy balance was the net energy between energy consumption during the operation of AD systems and energy recovery from methane production. The total energy consumption mainly included the energy demands of pumping, mixing and heating. Detailed analysis using average parameter values is listed as follows.

The energy consumption of pumping (*E_p_*, kWh/kg VSS) can be worked out according to Eq. (S1):

$E_{p}=E_{p1}+E_{p2}+E_{p3}+E_{p4}$ Eq. (S1)

where *E_p1_*, *E_p2_*, *E_p3_* and *E_p4_* are the pumping energy demands for mixed liquor recirculation, membrane permeate, sludge discharge and biogas recirculation, respectively (kWh/kg VSS). For the CAD reactor, the values of *E_p1_*, *E_p2_* and *E_p4_* are null.

*E_p1_*, *E_p2_*, *E_p3_* are calculated according to Eq. (S2)~Eq. (S4), respectively [^5^](#_ENREF_5):

$E_{p1}=\frac{Q_{1}\gamma h_{1}}{1000q\eta_{1}}$ Eq. (S2)

$E_{p2}=\frac{Q_{2}\gamma h_{2}}{1000q\eta_{1}}$ Eq. (S3)

$E_{p3}=\frac{Q_{3}\gamma h_{3}}{1000q\eta_{1}}$ Eq. (S4)

where *Q_1_* is the reactor recycle rate (5.9×10^-7^ m^3^/s for the AnDMBR), *γ* is 9800 N/m^3^, *h*_1_ is the measured hydraulic pressure head loss through the system (0.05 m for the AnDMBR), *q* is the influent VSS flow rate (3.1×10^-3^ kgVSS/h for the AnDMBR and 3.6×10^-5^ kgVSS/h for the CAD), *η_1_* is the pump efficiency (assuming 65%) [^6^](#_ENREF_6), *Q_2_* is the permeate flow rate (1.6×10^-7^ m^3^/s for the AnDMBR), *h_2_* is the head loss due to TMP and free water head (0.38+0.5=0.88 m for the AnDMBR in intermittent filtration mode), *Q*_3_ is the sludge discharge rate (4.0×10^-8^ m^3^/s for the AnDMBR and 2.9×10^-9^ m^3^/s for the CAD), and *h*_3_ is the estimated head loss due to sludge discharge (0.1 m).

In the AnDMBR reactor, *E_p4_*  can be calculated according to Eq. (S5) [^7^](#_ENREF_7):

$E_{p4}=\frac{MRT}{1000q(\lambda-1)\eta_{2}}\left[\left(\frac{p_{2}}{p_{1}}\right)^{1-\frac{1}{\lambda}}-1\right]$ Eq. (S5)

where *M* is the molar flow rate of biogas (4.5×10^-4^ mol/s), *R* is the gas constant (8.314 J/(mol K)), *T* is the biogas temperature (308 K), *P_1_* is the absolute inlet pressure (0.5 atm), *P_2_* is the absolute outlet pressure (1.0 atm), λ is the heat capacity ratio (1.3 for biogas) [^8^](#_ENREF_8), and *η_2_* is the gas pump efficiency (assuming 80%) [^7^](#_ENREF_7).

The energy consumption of mixing (*E_m_*, kWh/kg VSS) is calculated according to Eq. (S6):

$E_{m}=\frac{P_{m}V}{1000q}$ Eq. (S6)

where *P_m_* is the mixing power per unit volume of reactor (assuming 0.005 kW/m^3^) [^9^](#_ENREF_9), *V* is the reactor volume (69 L for the AnDMBR and 5 L for the CAD).

The energy consumption of heating (*E_h_*, kWh/kg VSS) is calculated according to Eq. (S7):

$E_{h}=\frac{C_{p}m(T_{R}-T_{0})}{3600q}$ Eq. (S7)

where *C_p_* is the specific heat of the sludge (4.2 kJ/(kg K)), *m* is the feed sludge rate (0.71 kg/h for the AnDMBR and 0.01 kg/h for the CAD), *T_R_* is the reactor temperature (308 K), and *T_0_* is the feed sludge temperature (298 K).

Energy can be recovered by methane combustion. The energy recovery of methane combustion (*E_r_*, kWh/kg VSS) can be calculated by Eq. (S8):

$E_{r}=\frac{E_{me}Q_{me}\eta_{3}}{24000q}$ Eq. (S8)

where *E_me_* is the methane energy potential (11 kWh/m^3^ CH_4_) [^10^](#_ENREF_10), *Q_me_* is the methane production (10.2 L/d for the AnDMBR and 0.01 L/d for the CAD), and *η_3_* is the conversion efficiency from methane energy into electricity (35%) [^10^](#_ENREF_10).

Energy balance analysis result is shown in Supplementary Fig. S7. Assuming that 2000 kg dry sludge per day is produced in a full-scale wastewater treatment plant and volatile solid content of the sludge is 0.6, the AnDMBR technology would lead to the annual net energy consumption (including energy recovery) reduction of ~6.6×10^5^ kWh compared to the CAD process.

Figure S1. Methane accumulation in BMP test

Figure S2. Ammonium concentrations in the reactors. Error bars represent the standard deviations (n=30).

Figure S3. Particle size distribution of AnDMBR sludge and effluent samples. Effluent 1 and Effluent 2 denote permeate samples during continuous and intermittent filtration mode, respectively.


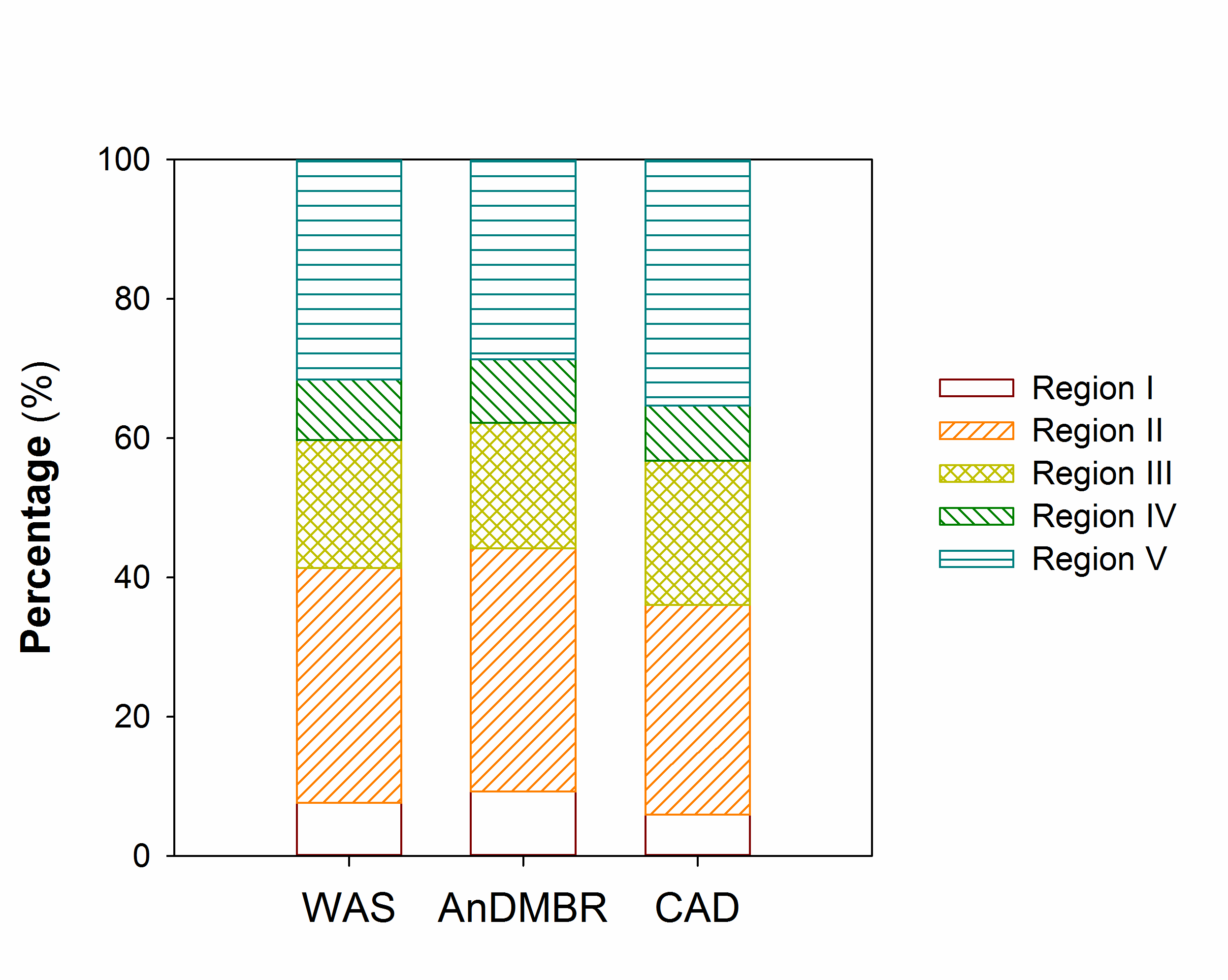


Figure S4. FRI analysis of different DOM samples. EEM peaks were divided into five regions, including simple aromatic proteins such as tyrosine and tryptophan (Regions I and II), fulvic acid-like substances (Region III), soluble microbial by-product-like materials (Region IV), and humic acid-like organics (Region V) [^11^](#_ENREF_11).

Figure S5. CST_n_ values of the digested sludge. Error bars represent the standard deviations (n=4).


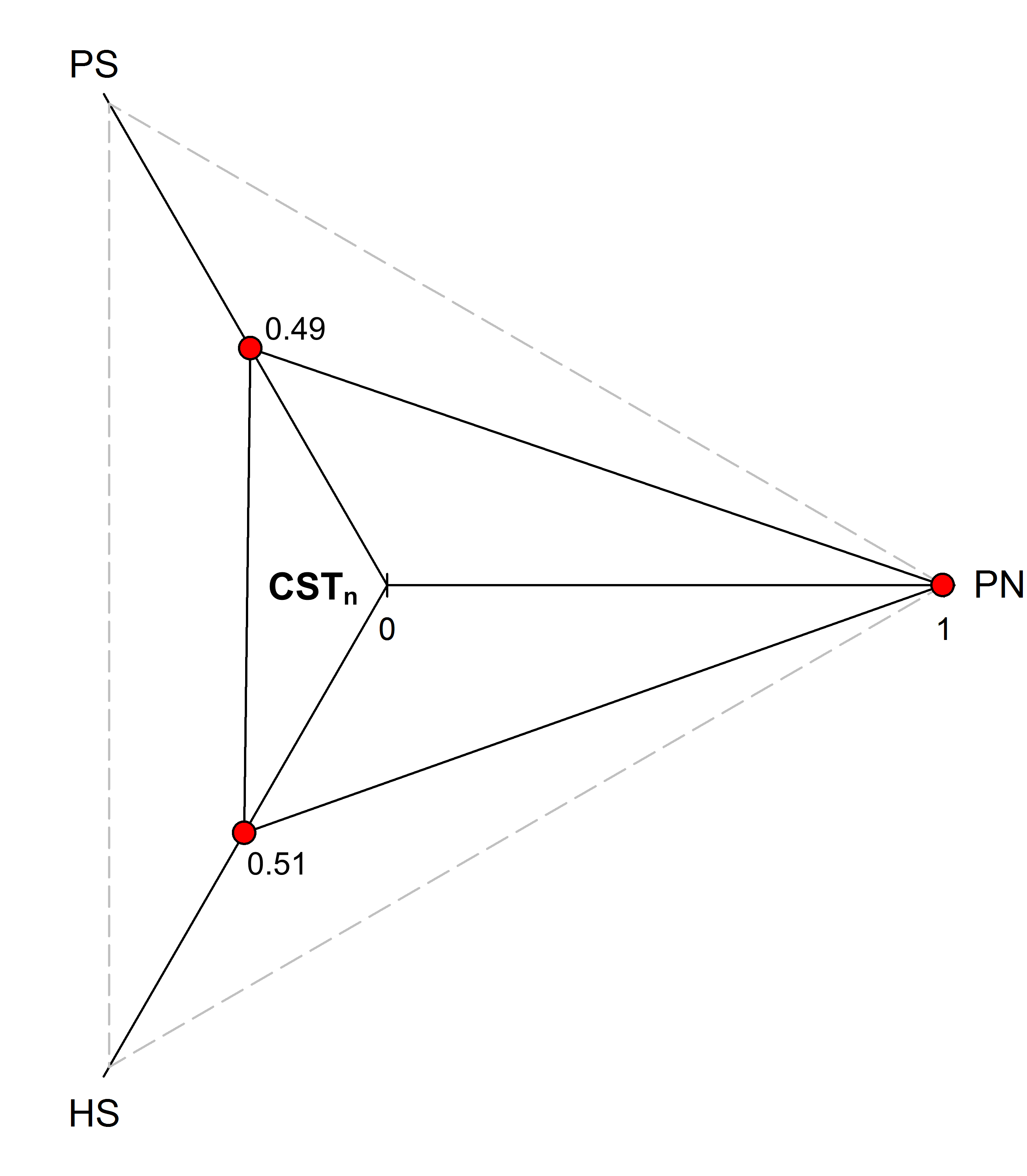


Figure S6. Correlation coefficients between CST_n_ values and various DOM composition contents. PS, PN and HS denote polysaccharides, proteins and humic substances, respectively


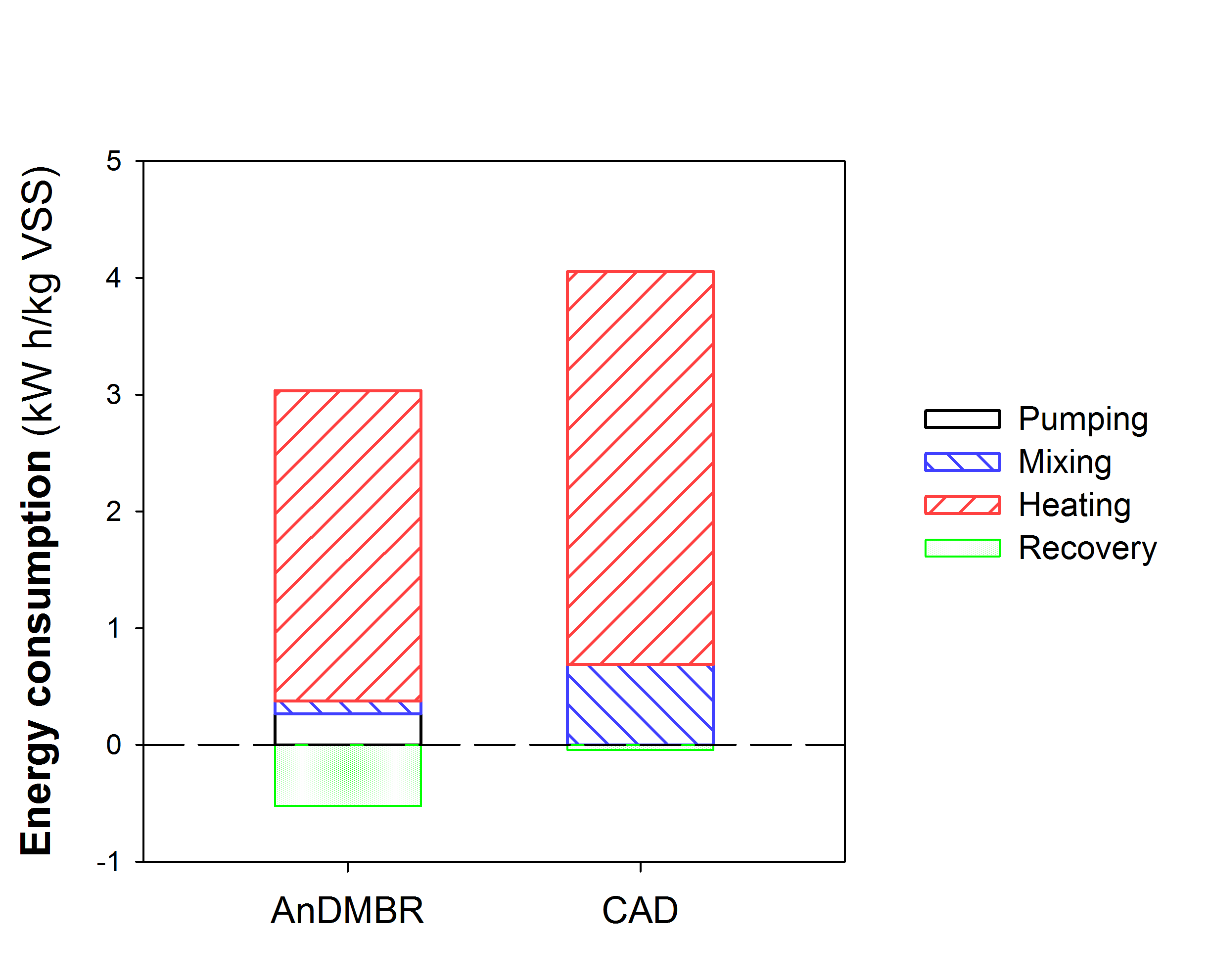


Figure S7. Energy balance of the two AD systems.

Table S1 Pyrosequencing and diversity statistics of bacterial and archaeal community.

| Bacterial community | | | | | | | | | | | |
| --- | --- | --- | --- | --- | --- | --- | --- | --- | --- | --- | --- |
| Sample | High-quality reads | | Rarefied reads | | OTU | | Chao | | Shannon | | Coverage |
| WAS | 9446 | | 4046 | | 407 | | 448 | | 5.15 | | 0.98 |
| AnDMBR | 8395 | | 4046 | | 360 | | 409 | | 4.95 | | 0.98 |
| CAD | 4046 | | 4046 | | 317 | | 317 | | 4.52 | | 1.00^b^ |
| Archaeal community | | | | | | | | | | | |
| Sample | High-quality reads | Rarefied reads | | OTU | | Chao | | Shannon | | Coverage | |
| WAS | 9567 | 8609 | | 45 | | 47 | | 1.53 | | 1.00^b^ | |
| AnDMBR | 12507 | 8609 | | 78 | | 80 | | 1.97 | | 1.00^b^ | |
| CAD | 8609 | 8609 | | 65 | | 67 | | 2.92 | | 1.00^b^ | |

^a^ Values were defined at a dissimilarity level of 0.03.

^b^ The number 1.00 denotes that the coverage value is larger than 0.999.

Table S2 SMA results based on different substrates.

| Substrate | SMA values ^a^ | |
| --- | --- | --- |
|  | AnDMBR | CAD |
| Acetate | 33.8 ± 2.3 | 10.0 ± 0.8 |
| H_2_/CO_2_ | 127.0 ± 3.8 | 48.3 ± 13.8 |

^a^ The data are given as mean value ± standard deviation (*n*=3).

References

1. Margulies M. *et al.* Genome sequencing in microfabricated high-density picolitre reactors. *Nature* **437**, 376-380 (2005).

2. Caporaso J. G. *et al.* QIIME allows analysis of high-throughput community sequencing data. *Nat Meth* **7**, 335-336 (2010).

3. Edgar R. C. UPARSE: highly accurate OTU sequences from microbial amplicon reads. *Nat Meth* **10**, 996-998 (2013).

4. Wang Q., Garrity G. M., Tiedje J. M., Cole J. R. Naïve Bayesian Classifier for Rapid Assignment of rRNA Sequences into the New Bacterial Taxonomy. *Appl. Environment. Microbiol.* **73**, 5261-5267 (2007).

5. Kim J. *et al.* Anaerobic Fluidized Bed Membrane Bioreactor for Wastewater Treatment. *Environ. Sci. Technol.* **45**, 576-581 (2011).

6. Wang Y. K., Sheng G. P., Shi B. J., Li W. W., Yu H. Q. A Novel Electrochemical Membrane Bioreactor as a Potential Net Energy Producer for Sustainable Wastewater Treatment. *Sci. Rep.* **3** (2013).

7. Pretel R., Robles A., Ruano M. V., Seco A., Ferrer J. The operating cost of an anaerobic membrane bioreactor (AnMBR) treating sulphate-rich urban wastewater. *Sep. Purif. Technol.* **126**, 30-38 (2014).

8. Martin I., Pidou M., Soares A., Judd S., Jefferson B. Modelling the energy demands of aerobic and anaerobic membrane bioreactors for wastewater treatment. *Environ. Technol.* **32**, 921-932 (2011).

9. Appels L., Baeyens J., Degreve J., Dewil R. Principles and potential of the anaerobic digestion of waste-activated sludge. *Prog. Energy Combust. Sci.* **34**, 755-781 (2008).

10. McCarty P. L., Bae J., Kim J. Domestic Wastewater Treatment as a Net Energy Producer-Can This be Achieved? *Environ. Sci. Technol.* **45**, 7100-7106 (2011).

11. Chen W., Westerhoff P., Leenheer J. A., Booksh K. Fluorescence Excitation−Emission Matrix Regional Integration to Quantify Spectra for Dissolved Organic Matter. *Environ. Sci. Technol.* **37**, 5701-5710 (2003).
